# Midwives’ empathy and shared decision making from women’s perspective - sensitivity of an assessment to compare quality of care in prenatal and obstetric care

**DOI:** 10.1186/s12884-022-05041-y

**Published:** 2022-09-20

**Authors:** Anja Alexandra Schulz, Markus Antonius Wirtz

**Affiliations:** 1 Department of Research Methods in the Health Sciences, University of Education Freiburg, Kunzenweg 21, 79117 Freiburg, Germany; 2 Department of Research Methods, University of Education Freiburg, Kunzenweg 21, 79117 Freiburg, Germany

**Keywords:** Midwifery care, Empathy, Shared decision making, Response shift, Structural equation modelling

## Abstract

**Background:**

For quality-oriented evaluation of prenatal and obstetric care, it is important to systematically consider the perspective of the women receiving care in order to comprehensively assess and optimize quality in a woman-centered manner. *Empathy* and *Shared Decision Making (SDM)* are essential components of woman-centered midwifery care. The aim of the study was to analyze measurement invariance of the items of the *Consultation and Relational Empathy (CARE)* and *Shared Decision Making-Questionnaire (SDM-Q-9)* scales depending on the prenatal versus obstetric care setting.

**Methods:**

One hundred fifty women retrospectively assessed aspects of woman-centered midwifery care in both prenatal and obstetric care setting. The birth of the child was a maximum of 12 months ago. A structural equation modelling approach was adopted to separate true effects from response shift (RS) effects depending on care setting. The latter were analyzed in terms of *recalibration* (changing women’s internal measurement standards), *Reprioritization* (changing associations of items and construct) as well as *Reconceptualization* (redefining the target construct).

**Results:**

A response shift model was identified for both assessments (pregnancy/birth: CFI = .96/.96; SRMR = .046/.051). At birth, both scales indicated lower quality of care compared with prenatal care (*SDM-Q-9-M/CARE-8-M*:|d| = 0.190/0.392). Although no *reconceptualization* is required for the items of both scales, RS effects are evident for individual items. Due to recalibration and reprioritization effects, the true differences in the items are partly underestimated (*SDM-Q-9-M/CARE-8-M*: 3/2 items) or overestimated (4/2 items).

**Conclusion:**

The structure of the constructs *SDM* and *Empathy*, indicating woman-centered midwifery care, are moderated by the care settings. To validly assess midwives’ empathy and shared decision making from women’s perspective, setting-dependent response shift effects have to be considered. The proven item-specific response effects contribute to a better understanding of construct characteristics in woman-centered care by midwives during pregnancy and childbirth.

**Supplementary Information:**

The online version contains supplementary material available at 10.1186/s12884-022-05041-y.

## Background

Midwife empathy and Shared Decision Making (SDM) are fundamental components of woman-centered midwifery care [1, 2]. For pregnant women, women giving birth and women in the postpartum period cared by midwives, the professional relationship with their midwife is an essential element in providing high quality professional midwifery care [3–5]. A good communication structure, trustful interaction, an emphatic and sensitive midwife, and participatory involvement in decision-making form the basis for this from the perspective of the women in care.

In Germany, midwives are assigned a central role (primary care provider) within an interprofessional woman-centered care context to ensure the regular course of pregnancy, birth and maternity (midwife-led continuity of care) [6]. By law, all women are entitled to continuous midwifery care during pregnancy, childbirth, postpartum and lactation (§ 134a Code of Social Law V). However, structural characteristics (e.g., shift work in clinical obstetrics) may lead to different midwives being responsible for the woman’s care. The range of services provided by midwives is multifaceted and includes independent and comprehensive counseling, care and monitoring of women. In addition, midwives are responsible for the autonomous management of physiological birth as well as the examination, care and monitoring of newborns and infants (§ 1 Midwifery Law). In case of deviations in the physiological course of pregnancy, birth and puerperium, midwives are obliged to consult physicians. The midwife’s presence during childbirth is required by law (§ 4 Midwifery Law).

Considering the midwife’s empathy, it is important to be aware that empathy has to be understood as a complex multidimensional construct [7], consisting of multiple components: (i) the emotional, (ii) the moral, (iii) the cognitive, and (iv) the behavioral components. Accordingly, empathy is a learnable, professional skill of communicating and is distinct from the view of a solely emotional experience on a subjective level [7]. It represents a key element in health care and is an important predictor of identifying women’s needs and fears as well as sharing information [8]. As a result, health outcomes are positively affected [9]. In the midwifery care context, the positive effect of empathy has been identified in terms of (i) empowering the woman for the upcoming birth, (ii) increasing the likelihood of positive birth experience and overall well-being, (iii) strengthening vital parameters (e.g., blood pressure), and (iv) enhancing pain management [10, 11].

Whereas clinical patient care has a strong focus on pathological aspects, midwives usually care for healthy women preventively who depend on the midwife’s emotional, communicative, interactive and physical support before and during the birth process [9]. Empathy skills enable the midwife to understand issues from the woman’s perspective. Childbirth represents an intense, powerful, and unpredictable life experience for the mother, which affects her subsequent life and daily routine [12]. Empathy is especially important when women do not want to speak in certain situations or when verbal communication is not possible (e.g., certain stages of birth) [10]. Thus, the nonverbal form of empathy (e.g., holding hands, intuitive gestures, and facial expressions) plays especially for the birth care setting a key role in the woman-centered care. This provides evidence that the construct structure and modes of expression of empathy in terms of critical indicators may vary across care settings [10].

SDM is a process in which the midwife, the woman, and her partner are in a trusting relationship. Hence, informed birth- and health-related decisions can be made interactively and consensually [13–15]. This process requires that (i) the midwife and the woman are able to define the problem or care options (e.g., mode of delivery), (ii) the best available evidence and the pros and cons (risks, benefits, costs) are known, (iii) the information is communicated to the woman in an understandable way, (iv) the woman’s values and preferences are identified and taken into account, and finally, (v) recommendations are made and a joint decision is made for further care [14, 16, 17]. Measuring SDM is considered a standard element of quality of care assessment [18]. Particularly in perinatal care, participatory decision making is considered a helpful element to prevent overmedicalization and inappropriate use of interventions during birth [19]. Evidence suggests that lack of involvement during decision making is associated with negative birth experiences [20, 21]. In contrast, women’s active role during SDM processes is associated with decreased perinatal depressive symptoms, lower likelihood of low birth weight, and preterm birth [22]. Especially in the stage of labor, the SDM process is challenging particularly due to the pressure of witnessing and unpredictable situational aspects that may affect the successful and low-risk birth process as much as possible [18]. In addition, it is unclear to what extent labor pain might override perceptions of interaction with the midwife during decision making and thus bias valid assessments of satisfaction with the SDM process [18].

Assessments measuring midwife empathy (e.g., Consultation and relational Empathy (CARE) scale [8, 23]) and SDM (e.g., SDM-Q-9-Questionaire [24]) can be used for comparative measurement across different care settings (pregnancy vs. birth). However, a mandatory prerequisite for valid comparisons is that responses on the Empathy and SDM scales are given against the same frame of reference in both care settings. Thus, the construct definition should remain constant across both settings. The association of the indicators with the latent construct (e.g., construct: empathy → indicator: active listening) should be identical in prenatal and obstetric care: Active listening should reflect the woman’s empathy experience in the same degree. This is not necessarily the case with self-reported assessments. If, for example, the positive birth experience affects the response to the individual items in the sense of a halo effect, although there is actually no objective difference with regard to the specific item content, the invariance assumption would be violated. Furthermore, women may prefer a different kind of decision making participation in the childbirth preparation phase than in the birth situation itself [25, 26]. When assessing differences in patient-reported experience of received care, different forms of invariance must be considered. For example, comparing scores at the time of birth care with baseline scores (care during pregnancy) may be misleading with regard to SDM, as the observed differences on item level may not reflect true difference in the SDM process on construct level across care settings [27].

Spranger and Schwartz [25, 26] distinguish three forms of response shift (RS): (i) *Recalibration* (changing women’s internal measurement standards): For example, because a woman may have had a particularly positive experience with the midwife during childbirth, her judgment may be somewhat more critical of prenatal care because of the new standard of comparison than she would have been without that experience. (ii) R*eprioritization* (changing women’s values): E.g., the woman perceives the construct empathy differently in the two care settings. In pregnancy, the cognitive-communicative aspects could be considered as the central element of empathy, whereas in childbirth the emotional closeness could be crucial for the construct. (iii) *Reconceptualization* (redefining the target construct): This would imply that in the birth situation, other aspects would represent as valid indicators of empathy than in prenatal care. E.g., in the birth situation it could be crucial that the midwife is sensitive to the specifics of the medical care context in the interaction with the woman, whereas this is considered obsolete in prenatal care.

These processes can lead to underestimation or overestimation of observed effects when comparing different care settings [28–30]. There are a variety of different methods for detecting RS [26]. In addition to the then-test (design approach), the structural equation modeling method of Oort et al. [28] is a widely used method to detect all three types of RS [31]. In addition, this method allows to consider the three different types of RS and to measure the true change or true difference [28].

Invariance measures are mainly used in the medical sector to assess the change in quality of life over the course of treatment from the patient’s perspective (e.g., [27, 29, 32]). To our knowledge, there are no studies in the maternity care setting that address RS in change measures related to indicators of woman-centered care. In addition, there are no RS studies for the CARE and SDM-Q-9 scales. There is evidence that women’s values and perceptions of self-reported experience may vary depending on the care setting (pregnancy, childbirth, postpartum) [33]. The study aimed to examine the response-shift for the Consultation and Relational Empathy (CARE) scale and the SDM-Questionnaire (SDM-Q-9) among pregnant women and women giving birth.

The analysis contributes to (i) improve knowledge on psychometric characteristics of accepted standards of patient-centered care, (ii) transfer and test the established CARE and SDM-Q-9 instruments in the field of midwifery care, and (iii) quantify the true difference and RS to all observed differences across care settings observed in the self-reported assessments on SDM and midwife empathy over the period of pregnancy care to birth care.

## Methods

### Study design and participants

Data were collected in a cross-sectional survey, which was carried out from June to July 2019. *N* = 2368 young families from a district in Germany received a written invitation and study information from the register of residents with a hyperlink to the digital questionnaire. Participant inclusion criteria comprised (1) majority age (≥ 18), (2) giving birth(s) in 2018, and (3) use of midwifery services during pregnancy and childbirth. *N* = 273 women (response rate 11.5%) chose to participate in the study. *N* = 150 women retrospectively completed the questionnaire 6 to 12 months after the child’s birth for both care settings, prenatal care, and care at childbirth. For *N* = 123 cases, data were only available for one of the two measurement time points and were excluded from data analysis. The questions referred to the experience of the women during pregnancy and childbirth. Because birth experience may result in biasing effects (e. g. Halo effects), retrospective assessments were used for both care settings [34]. The Ethics Committee of the German Society of Psychology classified the project as ethically acceptable (MAW 022019). All participants completed a digital informed consent form.

### Measures

#### 9-item Shared decision making-questionnaire (SDM-Q-9)

The generic SDM-Questionnaire (SDM-Q-9, German version) assesses the one-dimensional construct SDM in the interaction of midwives and women by 9 single items (Cronbach’s α = .94) [26]. These 9 individual items are scored on 6-point Likert scale from 0 = “Completely disagree” to 5 = “Completely agree”. Higher scores are associated with a higher degree of involvement of the woman in decision-making processes, e.g., “My midwife helped me to understand all the information.” (*SDM-M-5*). The instrument showed good psychometric properties in different clinical settings [35–37].

#### Consultation and relational empathy (CARE)

The validated German version of the Consultation and Relational Empathy (CARE; Cronbach’s α: .92–.94 [8, 23].) was used to assess midwives’ empathy in care. The original one-dimensional scale includes 10 items on emotional, moral, cognitive and behavioral aspects of midwives’ empathy from the perspective of the cared women (e.g., “Was the midwife interested in you as a whole person?” (*CARE-M-4*)). The women answered the items on a 5-point scale from 1 = “fully applies” to 5 = “does not apply at all”. Thus, low scores indicate high empathy. A previous study found that the last two items, which in contrast to the remaining items assess aspects of decision-making processes, had to be deleted in order to ensure the unidimensional of the assessment in the field of midwifery care [38] (see also [39]). Detailed information on the midwifery-specific adaptation of the CARE and SDM-Q-9 scales and the results of psychometric validation for the pregnancy setting (*N* = 201 mothers) have been published elsewhere [38]. Accordingly, only the 8 non-decision-related items were included in the analyses.

### Statistical analysis

#### Structural equation modelling

First, the transferability of the models valid for care in pregnancy [38] was tested confirmatory for the assessment of quality of care in childbirth. Measures of global and local model goodness of fit were used to assess model fit [40]. The main criteria were the root mean square error of approximation (RMSEA), the standardized root mean square residual (SRMR) and the incremental fit measures Tucker-Lewis Index (TLI) and Comparative Fit Index (CFI) [40]. Models with RMSEA < .05 exhibit good model fit because less than 5 percentage of the information in the variance-covariance matrix remains unexplained. RMSEA < .08 indicates acceptable fitting models [40]. The same reference values are valid for SRMR. If the TLI and CFI values are > .95, the model can be considered acceptable because more than 95% of the information that cannot be explained by the independence model (assumption: uncorrelated variables) is systematically explained [40]. Values > .97 indicate a good model fit. Indicator reliability (IR; sufficient: IR ≥ .40), factor reliability (FR; sufficient: FR ≥ .60), and average explained variance (AVE; sufficient: AVE ≥ .50) were used as measures of convergent local fit [40]. To ensure psychometrically solid separability of the constructs, the Fornell-Larcker criterion was considered [41]. This requires that each construct shares more variance with its own indicators than with the other model constructs.

The analysis was performed in 4 steps following Oort [28, 42]. (1) *Baseline model testing*: The measurement models are related to each other without parameter restriction. Insufficient model fit indicates situation-specific structure of the construct that cannot be transferred from pregnancy to birth (*reconceptualization*). (2) *Zero model*: Completely restricted models (loadings, intercepts, error variances) between the compared situations (pregnancy vs. birth). By means of a χ^2^-difference test it is examined whether there is complete measurement invariance or no RS at all. A significant test result indicates that further steps of RS detection are required. (3) *Selective release of model restrictions*: All factor loadings, all intercepts, and all measurement error variances were released separately, resulting in parameter-specific nested model comparisons. The violation of invariance was tested by χ^2^ -difference test. Inhomogeneities of factor loadings indicate *reprioritization*, inhomogeneities of intercepts indicate *uniform recalibration*, and inhomogeneities of error variances indicate *non-uniform recalibration*. (4) *Definition of the final RS model*: All inhomogeneities and parameter restriction releases identified as relevant in step 3 are integrated into a common model. This model can be used to determine the true or RS-adjusted difference [28]. Cohen’s d is calculated as effect size measure (d = 0.2, 0.5 and 0.8 are regarded as “small”, “medium” and “large”, respectively [43]).

Descriptive statistics as well as mean value comparisons (t-tests) were performed using SPSS 26.0. Structural equation models were estimated using the maximum likelihood algorithm estimation implemented in the software Amos 26.0 [44]. To avoid systematic bias due to missing data, missing values were imputed using expectation-maximization algorithm [45].

## Results

Of the 2368 young families contacted, a total of 273 persons (11.5%) completed the survey. Data for both pregnancy and birth care were available for 160 cases (58.6%). After excluding cases with missing values > 5 in the 17 scale items (> 16% missing values), *N* = 150 complete cases for both care settings were included in the analysis. The remaining missing values on the scale items were imputed using the EM algorithm [46]. The descriptive statistics of the analysis sample are presented in Table 1.

**Table 1:** Characteristics of the sample (*N* = 150)

	M	S.D.
Age	32.6	3.7
	Frequencies (n)	(%)
**Age**		
< 30 years	30	20.0
30–35 years	85	56.7
> 35 years	35	23.3
**Nationality**		
German	146	97.3
Another nationality	4	2.7
**Education**		
Secondary (general) & specialized school	69	46.0
Grammar or high school	79	52.7
Other	2	1.3
**Completed vocational training, higher education**		
Apprenticeship	35	23.3
Vocational school	25	16.7
Technical school	26	17.3
Engineering school	2	1.3
University, college	57	38.0
Other	5	3.3
**Marital status**		
Married, lives with spouse	115	76.7
Separated/divorced/widowed/single mother	35	23.3
**Insurance status**		
Statutory insurance	130	86.7
Private insurance	20	13.3
**Net-household income (monthly)**		
500 to less than 2000 €/month	20	13.3
2000 to less than 5, 000 €/month	115	76.7
≥ 5000 €/month	15	10.0
**Birth experience**		
Primipara	90	60.0
Two or more children born	60	40.00
**Premature birth**		
Yes	7	4.7
No	143	95.3
**High-risk pregnancy**		
Yes	27	18.0
No	120	80.0
I don’t know	3	2.0
**Mode of childbirth**		
Vaginal spontaneous birth	112	74.7
Intended caesarean birth	8	5.3
Unscheduled caesarean section/emergency caesarean-section	30	20.0

*M* mean, *S.D.* standard deviation

### Identification of integrated cross-setting structural models for the SDM-Q-9-M and the CARE-8-M

First, the fit of the birth data was tested for the underlying model, which had already been shown to fit the pregnancy data appropriately [38]. For the birth data, moderate to satisfactory model fit was demonstrated for both scales (*SDM-Q-9-M*: CFI = .96, TLI = .95, RMSEA = .139, SRMR = .030; *CARE-8-M*: CFI = .93, TLI = .89, RMSEA = .195, SRMR = .041; Table 2). The analysis of residual correlations identified violations due to local item dependencies (medium to strong ≥ .39) for 3 item pairs of the CARE-8-M scale (CARE-M-2 “*letting tell your story*” & CARE-M-3 “*really listening*”; CARE-M-4 “*interested in you as whole person*” & CARE-M-6 “*showing care and compassion*”; CARE-M-1 “*making you feel at ease*” & CARE-M-7 “*being positive*”). For the SDM-M scale the two item pairs (SDM-M-7 “*joint considerations of options*” & SDM-M-9 “*agreement for further care*”; SDM-M-8 “*joint selection of the option*” & SDM-M-9 “*agreement for further care*”) proved to be local dependent. These item pairs showed stronger associations with each other in the birth setting than in the pregnancy care setting. Nevertheless, these items exhibited high item-construct associations, which is consistent with the assumed unidimensional structure. After considering this model modifications, good model fit was achieved for both pregnancy and birth setting (integrated model pregnancy/birth: *SDM-Q-9-M*: CFI = .99/.98, TLI = .98/.97, RMSEA = .084/.103, SRMR = .018/.021; *CARE-8-M*: CFI = .97/.97, TLI = .94/.95, RMSEA = .125/.129, SRMR = .034/.032; Table 2).

**Table 2:** Measures of global fit for all estimated single CFA-models (*N* = 150)

	χ^**2**^	df	χ^**2**^/df	***p***	CFI	TLI	RMSEA [90%-CI]	SRMR
Acceptable fit threshold			< 3		≥ .95	≥ .95	≤ .08	
Good fit threshold			< 2	≥.05	≥. 97	≥ .97	≤ .05	≤ .08
**SDM-Q-9-M(idwifery)**								
Pregnancy model	74.80	24	3.12	≤ .001	.98	.97	.103 [.080; .130]	.025
Birth data (pregnancy model)	93.39	24	3.89	≤ .001	.96	.95	.139 [.110; .170]	.030
Modified model for birth data	56.93	23	2.48	≤ .001	.98	.97	.100 [.067; .132]	.021
Integrated SDM-Q-9-M Model (pregnancy | birth)	44.99 | 56.70	22	2.05 | 2.58	≤ .001	.99 | .98	.98 | .97	.084 [.048; .119] | .103 [.070; .136]	.018 | .021
Overall model integrated SDM-Q-9-M-model for pregnancy and birth	265.51	115	2.31	≤ .001	.96	.95	.094 [.079; .109]	.045
Zero-Model	351.41	140	2.51	≤ .001	.94	.94	.100 [.087; .113]	.050
Response-Shift Model SDM-Q-9-M	283.95	130	2.19	≤ .001	.96	.95	.089 [.075; .103]	.046
**CARE-8-M**								
Pregnancy model	57.59	19	3.03	≤ .001	.96	.95	.117 [.083; .152]	.033
Birth data (pregnancy model)	127.11	19	6.69	≤ .001	.93	.89	.195 [.164; .228]	.041
Modified birth model	55.92	16	3.50	≤ .001	.97	.95	.129 [.093; .167]	.032
Integrated CARE-8-M model (pregnancy | birth)	53.12 | 55.92	16	3.32 | 3.50	≤ .001	.97 | .97	.94 | .95	.125 [.089; .163] | .129 [.093; 0.167]	.034 | .032
Overall model integrated CARE-8-M-model for pregnancy and birth	161.32	87	1.85	≤ .001	.97	.96	.076 [.057; .094]	.048
Zero-Model	373.84	109	3.43	≤ .001	.89	.88	.128 [.114; .142]	.063
Response-Shift Model CARE-8-M	186.61	100	1.87	≤ .001	.97	.96	.076 [.059; .093]	.051

*CFI* Comparative Fit Index, *TLI* Tucker-Lewis Index, *RMSEA* Root Mean Square of Approximation, *SRMR* Standardized Root Mean Square Residual

### Detecting RS from pregnancy care to birth care

Step1: Satisfactory model fits were confirmed for the unrestricted baseline models in which the measurement models are estimated simultaneously for both care settings (overall model; *SDM-Q-9-M/CARE-8-M*: CFI = .96/.97, TLI = .95/.96, RMSEA = .094/.076, SRMR = .045/.048; Table 2). All items indicated high corrected item-total correlations (r_it,c_ = .72 to.93; Table 3).

**Table 3:** Descriptive statistics and measures of local fit for the CFA of the RS-scale structure (*N* = 150)^a^

	M	S.D.	***r***_**it,c**_	IR	
**SDM-Q-9-M** *(0 = completely disagree all, 5 = completely agree)*					
SDM-M-1 – has expressly informed that a decision must be taken	3.64 | 3.88	1.51 | 1.48	.73 | .74	.51 | .49	FR: .96 | .97 AVE: .74 | .78 α: . .97 | .97
SDM-M-2 – desired participation in decision making	3.73 | 3.67	1.36 | 1.54	.85 | .88	.70 | .75	
SDM-M-3 – information different options	4.15 | 3.68	1.32 | 1.65	.90 | .92	.87 | .92	
SDM-M-4 – explanation assets & drawbacks of the options	3.95 | 3.51	1.43 | 1.62	.93 | .91	.92 | .88	
SDM-M-5 – helped to understand all information	4.23 | 3.83	1.26 | 1.45	.84 | .90	.77 | .81	
SDM-M-6 – asked which option I preferred	4.03 | 3.73	1.44 | 1.62	.91 | .93	.92 | .94	
SDM-M-7 – joint consideration of options	3.79 | 3.29	1.49 | 1.68	.88 | .91	.77 | .82	
SDM-M-8 – joint selection of the option	3.53 | 3.43	1.60 | 1.67	.80 | .88	.62 | .74	
SDM-M-9 – agreement for further care	3.93 | 3.41	1.53 | 1.75	.85 | .83	.70 | .66	
**CARE-8-M** *(1 = fully applies, 5 = does not apply at all)*					
CARE-M-1 – making you feel at ease	1.22 | 1.47	.59 | .83	.79 | .86	.68 | .76	FR: .94 | .96 AVE: .67 | .77 α: .94 | .96
CARE-M-2 – letting you tell your “story“	1.24 | 1.81	.56 | 1.08	.82 | .85	.74 | .71	
CARE-M-3 – really listening	1.31 | 1.71	.59 | .97	.81 | .91	.67 | .84	
CARE-M-4 – being interested in you as whole person	1.26 | 1.81	.57 | 1.07	.88 | .83	.89 | .76	
CARE-M-5 – fully understanding your concerns	1.36 | 1.71	.68 | .98	.72 | .91	.61 | .81	
CARE-M-6 – showing care and compassion	1.30 | 1.55	.66 | .84	.89 | .90	.84 | .90	
CARE-M-7 – being positive	1.28 | 1.49	.66 | .87	.78 | .85	.54 | .76	
CARE-M-8 – explaining things clearly	1.36 | 1.63	.75 | .92	.73 | .82	.44 | .68	

^a^ 1st value: pregnancy, 2nd value: birth; Critical Ratio (C. R.) for all items ≥9; *M* mean, *S.D* standard deviation, *r*_*it,c*_ corrected item-total correlation, *IR* indicator reliability, *FR* factor reliability, *AVE* average variance extracted, *α* Conbach’s aplpha

Both scales proved to be highly reliable in both settings (α = .94 to.97; FR = .94 to.97). The latent constructs account for a high proportion of the variance in all individual items (AVE = .67 to.78) and were found to be highly separable according to the Fornell-Larcker criterion (max. latent correlation: .71 < min. square root of AVE: .82; Table 4).

**Table 4:** Intercorrelations of the scales and relevant scale properties for the pregnancy and birth setting (*N* = 150)

Scales	SDMS-Q-9-M	CARES-8-M	SDMG-Q-9-M	CAREG-8-M	α	M	S.D.	Skewness
**SDMS-Q-9-M**	***.86***^***1)***^	.51^***2)^	.40^*** 2)^	.14 ^2)^	.97	3.89	1.27	−1.37
**CARES-8-M**	.49^***3)^	***.82***^***1)***^	.20^** 2)^	.19^* 2)^	.94	1.29	0.54	−2.59
**SDMG-Q-9-M**	.37^***3)^	.16^*3)^	***.88***^***1)***^	.62^*** 2)^	.97	3.60	1.45	−1.02
**CAREG-8-M**	.18^*3)^	.20^*3)^	.71^***3)^	***.87***^***1)***^	.96	1.65	0.84	−1.50

^*)^ *p* < .05; ^**)^ *p* < .01; ^***)^ *p* < .001

^1)^ Values in the diagonal: Square root of average variance extracted (AVE)

^2)^ Values above the diagonal: Bivariate correlation of the scales

^3)^ Values below the diagonal: Bivariate correlation of the latent constructs

Step 2: The zero model, in which all model parameters are assumed to be invariant of the setting (cross-setting measurement invariance), showed a significantly worse data fit for *SDM-Q-9-M* and *CARE-8-M*, respectively (χ^2^/df_overall_ = 2.31/1.85; χ^2^/df_zero_ = 2.51/3.43; Table 2). Thus, for both constructs no measurement invariance is given, so that in the subsequent steps significant RS-effects are analyzed by means of a comprehensive RS model.

Step 3: To identify RS effects, each parameter that was restricted as invariant between the pregnancy and birth setting was successively released. The significance of removing each equating restriction was tested in a nested model comparison using the χ^2^-difference test.

For the SDM-Q-9-M scale, ten of the 27 parameters showed a significant difference between the two care settings (Table 5): Two factor loadings (RS-type: *reprioritization*; higher value at birth: *SDM-M-3: information about different options*; higher value at pregnancy*: SDM-M-1: informed that a decision must be taken*), five intercepts (RS-type: *uniform recalibration*; higher value at birth*: SDM-M-1*; *SDM-M-6: asked which option I preferred*; *SDM-M-8: joint selection of the option*; higher value at pregnancy: *SDM-M-7: joint considered options; SDM-M-9: agreement of further care*) and three error variances (RS-type: *non-uniform recalibration*; higher value at birth: *SDM-M-4: explanation assets & drawbacks of the options*; *SDM-M-9: agreement for further care*; higher value at pregnancy*: SDM-M-8: joint selection of the option*). Although the RS model, in which all RS-related violations are removed simultaneously, has additional 15 degrees of freedom, the global fit criteria actually indicate a slightly better model fit than the much less parsimonious overall model (CFI = .96, TLI = .95, RMSEA = .089, SRMR = .046; Table 2).

**Table 5:** Estimation of response shift parameters for the SDM-Q-9-M scale (*N* = 150)

	***Factor loadings***				***Intercepts***				***Error variance***				Overall^a^	
Item	FL	S.E.	***∆x***^**2b**^	***p***	IN	S.E.	***∆x***^**2b**^	***p***	VAR(e)	S.E.	***∆x***^**2b**^	***p***	***∆x***^**2b**^	***p***
SDM-M-1	0.949 *1.067 | 0.907*	.081 *.009 |.090*	4.457	**.035**	3.903 *3.784 | 4.074*	.101 *.115 | .111*	9.370	**.002**	1.107	.093	0.391	.532	12.526	**.006**
SDM -M-2	1.148	.079	1.524	.217	3.889	.101	0.823	.364	0.557	.049	0.013	.911	2.676	.444
SDM -M-3	1.288 *1.212 | 1.392*	.081 *.080 | .092*	10.520	**≤ .001**	4.142	.106	2.581	.108	0.229	.026	1.587	.208	14.589	**.002**
SDM -M-4	1.353	.084	3.261	.071	3.959	.112	0.408	.523	0.235 *0.166 | 0.318*	.027 *.028 | .047*	8.989	**.003**	14.265	.**003**
SDM -M-5	1.122	.075	0.715	.398	4.213	.097	1.674	.196	0.382	.035	0.341	.559	2.585	.460
SDM -M-6	1.378	.084	2.112	.146	4.074 *4.053 | 4.164*	.114 *.115 | .116*	4.850	**.028**	0.164	.021	0.125	.724	6.692	.082
SDM -M-7	1.325	.087	0.090	.764	3.712 *3.813 | 3.714*	.117 *.121 | .123*	7.947	**.005**	0.515	.047	0.964	.326	9.196	**.027**
SDM -M-8	1.239	.088	0.651	.420	3.690 *3.545 | 3.827*	.114 *.127 | .122*	17.149	**≤ .001**	0.838 *0.949 | 0.713*	.072 *.115 | .085*	4.632	**.031**	21.666	**≤ .001**
SDM-M-9	1.255	.089	0.082	.775	3.846 *3.946 | 3.812*	.117 *.121 | .133*	4.296	**.038**	0.894 *0.684 | 1.066*	.078 *.085 | .128*	8.229	**.004**	13.086	**.004**

*FL* factor loading, *IN* intercept, *e* error variance, *S.E.* standard error, *df* degrees of freedom, ***∆x***^**2**^= on release of parameter; Critical Ratio (C.R.) for all items ≥ 9.0

^a^ = FL, IN and e are freely estimated for each item simultaneously; In case of RS (*p* ≤ .05), a second, italicized row was generated with estimated parameters (1st value: pregnancy, 2nd value: birth); ^b^ df_diff_ for all model comparisons = 1

For the *CARE-8-M* scale, RS-differences between the two care settings were evident for nine of the 24 parameters (Table 6). *Reprioritization* was present for three items (higher loading at birth: *CARE-M-2: letting you tell your story*, *CARE-M-4: being interested as whole person*; higher loading at pregnancy: *CARE-M-6: showing care and compassion*). *Uniform recalibration* was found for three items *CARE-M-2*, *CARE-M-4* (both higher intercept at birth), and *CARE-M-6* (higher intercept at pregnancy). *Non-uniform recalibration* was detected for the items *CARE-M-1: making you feel at ease*, *CARE-M-2*, and *CARE-M-4* due to higher error variances at birth. Global measures of goodness of fit indicate the more parsimonious comprehensive RS model to fit as well as the unrestricted overall model (CFI = .97, TLI = .96, RMSEA = .076, SRMR = .051; Table 2).

**Table 6:** Estimation of response shift parameters for the CARE-8-M scale (*N* = 150)

	***Factor loadings***				***Intercepts***				***Error variance***				Overall^a^	
Item	FL	S.E.	***∆x***^**2b**^	***p***	IN	S.E.	***∆x***^**2b**^	***p***	VAR(e)	S.E.	***∆x***^**2b**^	***p***	***∆x***^**2b**^	***p***
CARE-M-1	0.460	.033	1.399	.237	1.210	.043	1.011	.315	.152 *.101 | .175*	.014 *.013 | .022*	9.638	**.002**	13.672	**.003**
CARE-M-2	0.560 *0.488 | 0.551*	.040 *.037 | .046*	7.937	**.005**	1.330 *1.244 | 1.433*	.054 *.046 | .067*	11.907	***≤*.001**	.214 *.083 | .324*	.020 *.011 | .040*	43.864	**≤.001**	63.435	**≤ .001**
CARE-M-3	0.531	.038	2.352	.125	1.337	.051	0.154	.695	.141	.013	1.188	.276	3.747	.290
CARE-M-4	0.566 *0.539 | 0.571*	.039 *.035 | .048*	14.464	***≤*.001**	1.319 *1.260 | 1.442*	.053 *.047 | .071*	12.853	***≤*.001**	.154 *.036 | .268*	.016 *.008 | .037*	73.908	**≤.001**	85.661	**≤ .001**
CARE-M-5	0.536	.039	0.800	.371	1.366	.052	0.330	.565	.181	.016	2.303	.129	3.930	.269
CARE-M-6	0.512 *0.611 | 0.485*	.035 *.042 | .036*	27.667	***≤*.001**	1.270 *1.300 | 1.244*	.047 *.055 | .051*	7.479	**.006**	.072	.009	0.035	.851	33.966	**≤ .001**
CARE-M-7	0.463	.034	0.991	.319	1.244	.047	3.053	.081	.182	.016	0.669	.413	5.051	.168
CARE-M-8	0.472	.038	2.536	.111	1.338	.053	0.098	.754	.280	.024	0.271	.603	3.229	.358

*FL* factor loading, *IN* intercept, *e* error variance, *S.E.* standard error, *df* degrees of freedom; ***∆x***^**2**^= on release of parameter; Critical Ratio (C.R.) for all items ≥ 9.0

^a^ = FL, IN and e are freely estimated for each item simultaneously; In case of RS (*p* ≤ .05), a second, italicized row was generated with estimated parameters (1st value: pregnancy, 2nd value: birth); ^b^ df_diff_ for all model comparisons = 1

Step 4: Based on the RS model, the contribution of the RS to the observed difference between care settings was determined [30]. The estimate of the true item-specific difference results from the assumption that the measurement models in pregnancy care can be transferred to birth care (setting-invariant measurement models). At the item level, the standardized true difference of pregnancy versus birth for the *SDM-Q-9-M* scale ranged from d_true_ = − 0.184 to − 0.240 (Table 7). For the *CARE-8-M* scale, the standardized true difference of the midwife’s empathy of pregnancy compared with birth ranged from d_true_ = 0.265 to 0.385 (Table 8).

**Table 7:** Evaluation of RS and true difference for SDM-Q-9-M scale from pregnancy to birth (*N* = 150)

SDM-Q-9-M	Type of RS	Pregnancy		Birth					Effect-size^a^				
Item		$\overline{\boldsymbol{x}}$	S.D.	$\overline{\boldsymbol{x}}$	S.D.	*p*	*r*	Mean difference	Observed difference (d)	‘True’ difference (d)	RS total (d)	RS Recali-bration (d)	RS Reprioritization (d)
SDM-M-1	reprioritization, uniform recalibration	3.64	1.51	3.88	1.48	.094	.34*	0.24	0.134	−0.184	**0.318**	0.290	0.028
SDM-M-2		3.73	1.36	3.67	1.54	.646	.35*	−0.06	−0.036				
SDM-M-3	reprioritization	4.15	1.32	3.68	1.65	***≤***.001	.34*	−0.47	−0.270	−0.216	**−0.054**	−0.022	−0.032
SDM-M-4	non-uniform recalibration	3.95	1.43	3.51	1.62	.003	.34*	−0.44	−0.252	−0.240	**−0.012**	−0.012	n. a.
SDM-M-5		4.23	1.26	3.83	1.45	***≤***.001	.40*	−0.40	−0.256				
SDM-M-6	uniform recalibration	4.03	1.45	3.73	1.62	.040	.31*	−0.30	−0.166	−0.236	** 0.070**	0.070	n. a.
SDM-M-7	uniform recalibration	3.79	1.49	3.29	1.68	***≤***.001	.32*	−0.50	−0.261	−0.214	**−0.047**	−0.047	n. a.
SDM-M-8	non-uniform recalibration with intercept influence	3.53	1.60	3.43	1.67	.508	.44*	−0.10	−0.058	−0.222	** 0.164**	0.164	n. a.
SDM-M-9	non-uniform recalibration with intercept influence	3.93	1.53	3.41	1.75	***≤***.001	.34*	−0.52	−0.265	−0.199	**−0.067**	−0.067	n. a.
Total		3.89	1.27	3.60	1.45	.023	.40*	−0.29	−0.190				

^a^For effect size calculation, the estimated parameters of the response shifts model CARE-8-M/SDM-Q-9-M were divided by the estimated standard deviation; d = Cohen’s d effect size; Values of 0.2, 0.5, 0.8 indicate “small”, “medium” and “large” effect-sizes; *n. a.* not applicable. Please note that rounding errors may occur when summing the effect sizes for the true difference and the response shift. ^*^ *p* < .01

**Table 8:** Evaluation of RS and true difference for CARE-8-M scale from pregnancy to birth (*N* = 150)

CARE-8-M	Type of RS	Pregnancy		Birth						Effect-size^a^			
item		$\overline{\boldsymbol{x}}$	S.D.	$\overline{\boldsymbol{x}}$	S.D.	*p*	*r*	Mean difference	Observed difference (d)	‘True’ difference (d)	RS total (d)	RS Recali-bration (d)	RS Reprioriti-zation (d)
CARE-M-1	non-uniform recalibration	1.22	0.59	1.47	0.83	.002	.09	0.25	0.239	0.282	**−0.042**	−0.042	n. a.
CARE-M-2	reprioritization, non-uniform recalibration with intercept influence	1.24	0.56	1.81	1.08	***≤***.001	.08	0.57	0.484	0.265	** 0.219**	0.185	0.034
CARE-M-3		1.31	0.59	1.71	0.97	***≤***.001	.17*	0.40	0.372				
CARE-M-4	reprioritization, non-uniform recalibration with intercept influence	1.26	0.57	1.81	1.07	***≤***.001	.12	0.55	0.459	0.288	** 0.171**	0.154	0.017
CARE-M-5		1.36	0.68	1.71	0.98	***≤***.001	.15	0.35	0.303				
CARE-M-6	reprioritization, uniform recalibration	1.30	0.66	1.55	0.84	***≤***.001	.23**	0.25	0.246	0.385	**−0.139**	−0.059	−0.079
CARE-M-7		1.28	0.66	1.49	0.87	.006	.25**	0.21	0.217				
CARE-M-8		1.35	0.75	1.63	0.92	***≤***.001	.25**	0.28	0.265				
Total		1.29	0.54	1.65	0.85	***≤***.001	.19*	0.36	0.392				

^a^For effect size calculation, the estimated parameters of the response shifts model CARE-8-M/SDM-Q-9-M were divided by the estimated standard deviation; d = Cohen’s d effect size; Values of 0.2, 0.5, 0.8 indicate “small”, “medium” and “large” effect-sizes; *n. a.* not applicable. Please note that rounding errors may occur when summing the effect sizes for the true difference and the response shift. ^*^ *p* < .05; ^**^ *p* < .01

RS corresponds to the difference between the true and the observed change. Positive (vs. negative) RS values indicate that the item-level difference is more positive (vs. more negative) than would be expected based on latent construct values. For the *SDM-Q-9-M* scale, four marginal RS effects point in the negative direction (d_RS_ = − 0.012 to − 0.067; Table 7). The items with negative RS indicate a more significant decrease in shared decision making from pregnancy compared to birth than would be expected based on the true difference. The three positive total RS effects are generally higher (d = 0.070 to 0.318). The effect is particularly pronounced for item *SDM-M-1: informed that a decision must be taken*, as a positive difference was measured despite the negative true difference (d_true_ = − 0.184; d_observ_ = 0.134). *Recalibration* is present for all items with RS. Only the items *SDM-M-1* and *SDM-M-3* shows *reprioritization* due to setting-specific associations of the item with the underlying construct (unstandardized factor loading).

For the *CARE-8-M* items, a violation of the invariance of the factor loading occurs for three of the eight items. Overall, the factor loadings are more dependent on the respective setting than for *SDM-Q-9-M* (*reprioritization*; CARE-M-2, CARE-M-4 and CARE-M-6; |d| = 0.017–0.079; Table 8). For example, the item *CARE-M-2*: *letting you tell your story* (pregnancy: FL = 0.488 vs. birth: FL = 0.551; Table 6) is significantly more highly associated with the latent construct in the birth setting. Together with the *recalibration* effects, which affect all items with RS, this results in total RS effects for all items. For the items *CARE-M-2* and *CARE-M-4* there were positive RS effects (d_RS_ = 0.219/ 0.171; Table 8). This means that the true difference is overestimated by these items. The remaining two items with RS (*CARE-M-1* and *CARE-M-6*) underestimate the true difference (d_RS_ = − 0.042 to − 0.139). When interpreting the values, it must be taken into account that high values on the *CARE-8-M* scale reflect a low level of empathy.

At the latent mean level, both the *SDM-Q-9-M* (Cohen’s d = 0.26, *p* < .001) and *CARE-8-M* (Cohen’s d = 0.37, *p* < .001) scales showed significantly worse scores for obstetric care compared to pregnancy care.

## Discussion

For both the CARE-M and SDM-Q-9-M scales, a subset of the items represent the underlying latent construct empathy and SDM, respectively, in a comparable and fair manner independent of the care setting. However, individual items indicate a stronger or weaker difference between care settings than should be expected based on differences at the latent construct level. Thus, in diagnostic application in maternity care practice, a distinction must be made between (i) which item information actually represents genuine empathy differences (true setting-dependent empathy differences) and (ii) which item information represents differences that dissociate from the overall empathy difference. This contributes to a setting-specific understanding of which behavioral and interactional aspects characterize the midwife’s empathy and SDM behavior from the women’s perspective.

The fact that the women perceive the empathy of the midwife during the birth situation as significantly weaker is reflected in all 8 items of the CARE-8-M scale. For the five empathy indicators “making you feel at ease” (CARE-M-1), “really listening (CARE-M-3), “fully understanding your concerns“ (CARE-M-5), “being positive“ (CARE-M-7), and “explaining things clearly” (CARE-M-8), there are no RS effects. Thus, these items validly represent the differences in equal strength. These empathy aspects, which mainly address the interactional-communicative behavioral component as well as the nonverbal empathy form, can thus be considered as setting-independent core aspects of midwifery empathy [1, 11]. For these items, it can be ruled out that (i) setting-specific Halo effects [47] might cause varying item-construct associations, (ii) a changing woman’s assessment standard might lead to an unexpected lower- or higher-than-average score, and (iii) that empathy perception might change structurally [28, 46].

“Letting you tell your story” (CARE-M-2) and “being interested in you as a whole person” (CARE-M-4), however, are rated significantly worse in the birth situation than would be expected based on the general empathy effect (mean difference ≥ .55, *recalibration*). This may be due to the fact that these two empathy aspects are difficult to implement by the midwife in direct birth care [33]. Perhaps the importance of these communication aspects attenuates during the birth process because women consider them to be less relevant [48]. Additionally, the variance of these two items is most significantly increased in the birth setting compared to the pregnancy setting (reduction of the ceiling effect). This increase in information variance is reflected in stronger item-construct association in the birth setting (*reprioritization*). This strengthens the association of the item information in CARE-M-2 and CARE-M-4 with the general empathy construct. Thus, the validity of the items increases because differences in the midwife’s empathic behavior are reflected in a more differentiated way due to the higher trait variance in the birth setting. The opposite effect appears for the item “showing care and compassion” (CARE-M-6). The difference in the item means between the settings is unexpectedly weak and the item variance increases the least. This is in line with the basic orientation of the salutogenetic professional ethos of midwives, in which the biopsychosocial health of women is the focus of practical action, and their rights and dignity are preserved respectfully and with compassion during this vulnerable period [1, 49]. After considering these RS effects, these three items also contribute to a more reliable and differentiated diagnosis of empathy-related differences.

SDM also scores significantly lower during birth care compared to pregnancy care. “Desired participation in decision making” (SDM-M-2), “explanation assets & drawbacks of the options” (SDM-M-4), and “helped to understand all information” (SDM-M-5), prove to be valid indicators independent of the setting context (no item-specific RS effects). Thus, the overall differences of SDM between care settings can be assessed fairly by using these three items [50]. Overall, RS effects are present for 6 SDM items. In contrast to the empathy scale, the RS effects for 5 of these 6 SDM items are considerably weaker (|d_RS_| = .012–.164). While the mean score of these five SDM items is significantly lower in the birth situation, the SDM aspect “has expressly informed that a decision must be taken” (SDM-M-1) is rated markedly better than expected. In fact, SDM-M-1 is the only one of the 9 SDM-items that measures higher scores in the birth setting. This does not validly represent the general difference in SDM between settings (*recalibration*). Accordingly, this increase in the mean must be considered an artifact due to the recalibration effect. The increase results exclusively from the fact that the women’s assessment standard for the birth setting changes. It is reasonable to assume that this communicative aspect is of higher importance to women during pregnancy than during childbirth. Particularly when caring for women who express great fear of childbirth, the participatory communication aspect is important in childbirth preparation to preventively mitigate fears and empower women regarding childbirth preparation [51]. During childbirth, women are particularly vulnerable and unanticipated health-related decisions must be made under time pressure [52]. In this situation, women are more dependent on the expertise and experience of health care providers for decision making than during pregnancy [53]. These setting-specific characteristics, as well as the positive birth experience itself, may result in a woman’s evaluation standard being more critical in pregnancy than for childbirth [33].

Overall, the detected RS effects are weak, as expected (except for SDM-M-1), because they are superimposed by the true differences and the different forms of RS (*reprioritization*, forms of *recalibration*) may influence each other [42]. However, significant RS should always be considered independently of the true differences for the analysis of the significance of differences in terms of content since they systematically reflect different difference components. In order to be able to depict the true differences in a valid way, considering RS separates theoretically significant difference components that can make the meaning of the construct or its setting dependent change identifiable [46]. RS indicates that, due to these differential components, the observed differences must not be interpreted as a homogeneous change in a characteristic that is erroneously assumed to be stable. The results illustrate the psychometric advantages of invariance testing in the assessment of patient-reported outcomes by means of self-assessments. This procedure should be established as a standard of analysis in the health care sector in order to provide fairness and validity of diagnostic assessments in different clinical contexts and settings [30].

The present study represents a central step in improving construct understanding of empathy and SDM in midwifery care regarding women’s experiences and in identifying valid construct indicators within the context of setting-specific characteristics. It becomes evident that woman-centered care elements such as Empathy and SDM may be less satisfactorily implementable in the birth setting than in prenatal care. This challenge is in line with existing research findings and highlights the need to assume that assessment behaviors are influenced by situational characteristics (e.g., labor, negative/positive birth experiences), limiting comparability across care settings [18, 33, 54].

In general, across both care settings, women appear to rate the midwife’s empathy better than the midwife’s SDM behavior. Although SDM effects are well documented in health services research and the concept is considered a central component of high-quality patient care, implementation in routine care is lacking [55]. SDM is often considered an add-on to discipline-specific care delivery in the care setting. Here, clarification is needed on whether the use of standardized procedures can simplify SDM processes and facilitate the efficiency of procedures to ensure women’s rights [55]. This marks a starting point for clinical practice to align maternity care more closely with women’s needs [56]. In addition, due to the complexity of the delivery system and increasing workload density in midwifery care, advancing professional education, and promoting interprofessional collaboration among providers within obstetric care is useful to improve SDM secondarily [56].

Furthermore, a deeper understanding of the diagnostic properties for the empathy construct as well as for the SDM process could be created by identifying valid and change-sensitive indicators suitable to fairly capture differences between care settings. As a result, the interactional-communicative as well as the nonverbal behavioral components of midwifery become more prominent in the empathy domain. In SDM, regardless of the care setting, support in information processing and clarification of individual participation preferences are central.

### Limitations

Data were collected retrospectively and for both care settings at one measurement point in time (6 to 12 months after birth). Accordingly, a biased memory (recall bias) or an insufficiently differentiated (halo effect) or too strongly contrasted judgment of the separate settings (contrast effects) cannot be excluded [57]. The data were collected in a district in southwestern Germany, so that their general representativeness for midwifery care is not given. The analysis of data from further parent samples would be desirable to critically examine the generalizability of the findings. Local dependencies of the items were considered to ensure construct validity for both settings (see Additional file 1). Although this is associated with uncertainties in the modeled covariance structure, it is in line with the recommended procedure for RS analysis [27, 42].

Furthermore, it has to be noted that despite the SEM approach, the RS analysis must be understood as at least partially exploratory [58]. The chosen analysis approach ensured that all significant moderation effects of the settings were considered in an integrated manner in the overall model. Hence, all decisions could be substantiated transparently, and all forms of RS could be determined. As an alternative to our procedure of full parameter-specific significance testing, the equally exploratory modification indices-based iterative procedure of “specification search” by Oort [28] or “Then” test [59] could be used. RS analysis can only identify which RS are present without being able to identify their causes [46]. To be able to test the significance of RS effects for the quality of obstetric care provided by midwives, theory-based structural models in which not only SDM and empathy but also their consequences are modeled should be empirically investigated [60]. Furthermore, theoretically relevant covariates should be considered or interventions with item-selective effect hypotheses (e.g., targeted promotion of emotional components of midwives’ empathy) should be tested. Also, other care settings, such as postnatal care or care after miscarriage, should be investigated in order to empirically analyze the interaction of setting characteristics and the *SDM-Q-9-M* and *CARE-8-M* measurement instruments.

## Conclusion

Empathy and SDM are of particular importance in care settings because they represent process characteristics that are essential determinants of primary treatment outcomes (patient-reported outcomes: e.g. quality of life, patient satisfaction, quality of care) [60–62]. The results indicate that the process-oriented instruments for measuring SDM (*SDM-Q-9-M*) and Empathy (*CARE-8-M*), which are widely accepted in patient-centered care [62], could be successfully transferred to the field of midwifery care. We were able to identify item-specific RS effects for both instruments. When these are taken into account, valid comparisons between care settings can be made [28, 46, 58]. In midwifery practice, the two instruments can be used to assess women’s views of their SDM and empathy needs in a setting-specific manner. Thus, the midwife’s professional role can be better understood in terms of the midwife-woman relationship [63]. The scales allow the identification of setting-specific strengths and weaknesses in everyday care in order to develop targeted measures for the promotion of midwifery competencies in terms of woman-centered qualitative care [64]. For this purpose, the results reveal relevant starting points for interventions: (i) Strengthen the focus on woman-centered care aspects in the birth setting, (ii) optimizing SDM processes independent of the care setting. In contrast to existing assessment tools (e.g., Midwifery Empathy Scale [64]), it is possible to use the present scales for both external and self-assessment (e.g. [63]). The systematic comparison results in a better understanding of perspective-related differences and results in improved midwife-woman interactions [65].

## Supplementary Information

**Additional file 1.** Measurement Model of the SDM-Q-9-M and CARE-8-M scales used in RS detection. Graphical representation of the confirmatory factor analysis of the SDM-Q9-M and CARE-8-M-scale and measures of the local model fit.

## Data Availability

The datasets created and analyzed as part of the current study are not publicly available, but can be obtained from the corresponding author (anja.schulz@ph-freiburg.de) upon reasonable request. The data are not made freely available to the public, as this was not explicitly requested in the ethics application.

## Abbreviations

AVE: Average variance extracted
CARE(− 8-M): Scale for measuring (the midwife’s) empathy
CFI: Comparative Fit Index
Cohen’s d_(observ/RS/true)_: Effect size measure for mean differences (observed effect / Response shift effect / true effect)
FR: Factor reliability
IR: Indicator reliability
r_it,c_: Corrected item-total correlation
RMSEA: Root Mean Square of Approximation
RS: Response shift
SDM-Q9(−M): Scale for measuring shared decision making (by the midwfe)
SEM: Structural equation modeling
SRMR: Standardized Root Mean Square Residual
TLI: Tucker-Lewis Index
VAR_(e)_: Variance of error term
