# Supplementary material for: Midwives’ empathy and shared decision making from women’s perspective - sensitivity of an assessment to compare quality of care in prenatal and obstetric care
Source: BMC Pregnancy Childbirth. 2022 Sep 20;22:717. doi: 10.1186/s12884-022-05041-y (PMC9487070; doi:10.1186/s12884-022-05041-y)
Supplement: Supplementary file 1 — Additional file 1. Measurement Model of the SDM-Q-9-M and CARE-8-M scales used in RS detection. Graphical representation of the confirmatory factor analysis of the SDM-Q9-M and CARE-8-M-scale and measures of the local model fit. [file 12884_2022_5041_MOESM1_ESM.pdf]

Additional file

Measurement Model of the SDM-Q-9-M scale used in RS detection

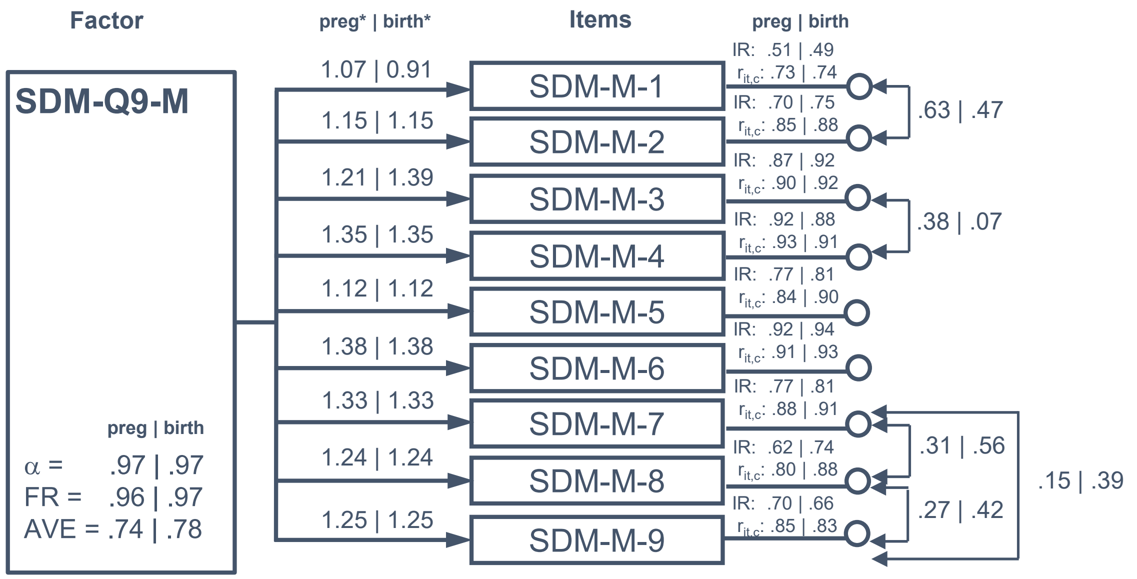

* unstandardized estimates; FR = factor reliability, AVE = average variance extracted, IR = indicator reliability, $r_{it,c}$=corrected item-total correlation, Critical Ratio for all items ≥ 9, α = Conbach´s alpha

Measurement Model of the CARE-8-M scale used in RS detection

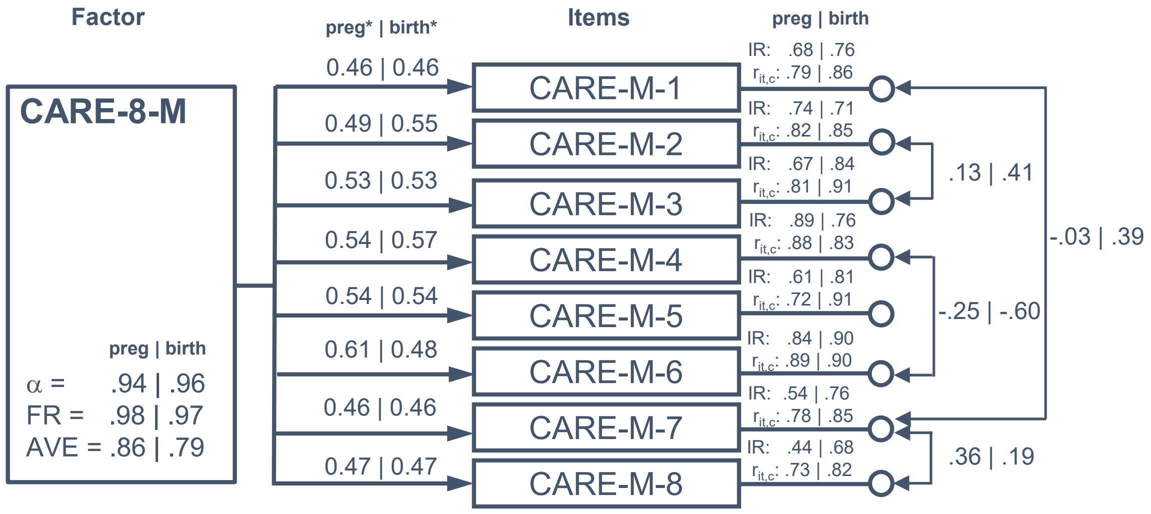

*unstandardized estimates; FR = factor reliability, AVE = average variance extracted, IR = indicator reliability, $r_{it,c}$=corrected item-total correlation, Critical Ratio for all items ≥ 9, α = Conbach´s alpha
